# The Dynamics of the Defense Strategy of Pea Induced by Exogenous Nitric Oxide in Response to Aphid Infestation

**DOI:** 10.3390/ijms18020329

**Published:** 2017-02-05

**Authors:** Agnieszka Woźniak, Magda Formela, Piotr Bilman, Katarzyna Grześkiewicz, Waldemar Bednarski, Łukasz Marczak, Dorota Narożna, Katarzyna Dancewicz, Van Chung Mai, Beata Borowiak-Sobkowiak, Jolanta Floryszak-Wieczorek, Beata Gabryś, Iwona Morkunas

**Affiliations:** 1 Department of Plant Physiology, Poznań University of Life Sciences, Wołyńska 35, 60-637 Poznań, Poland; agnieszkam.wozniak@gmail.com (A.W.); formelamagda@o2.pl (M.F.); p.bilman@o2.pl (P.B.); grzeskiewiczkasia@gmail.com (K.G.); chungmv@vinhuni.edu.vn (V.C.M.); florysza@up.poznan.pl (J.F.-W.); 2 Institute of Molecular Physics, Polish Academy of Sciences, Smoluchowskiego 17, 60-179 Poznań, Poland; Waldemar.Bednarski@ifmpan.poznan.pl; 3 Institute of Bioorganic Chemistry, Polish Academy of Sciences, Noskowskiego 12/14, 61-704 Poznań, Poland; lukasmar@ibch.poznan.pl; 4 Department of Biochemistry and Biotechnology, Poznań University of Life Sciences, Dojazd 11, 60-632 Poznań, Poland; dorna@o2.pl; 5 Department of Botany and Ecology, University of Zielona Góra, Prof. Z. Szafrana 1, 65-516 Zielona Góra, Poland; k.dancewicz@wnb.uz.zgora.pl (K.D.); b.gabrys@wnb.uz.zgora.pl (B.G.); 6 Department of Entomology and Environmental Protection, Poznań University of Life Sciences, Dąbrowskiego 159, 60-594 Poznań, Poland; borowiak@up.poznan.pl

**Keywords:** nitric oxide, edible pea, pea aphid, pisatin, flavonoids, semiquinone radicals, electrical penetration graph, population demographic parameters

## Abstract

The aim of this study was to investigate the effect of exogenous nitric oxide (NO), i.e., *S*-nitrosoglutathione (GSNO) and sodium nitroprusside (SNP), on the metabolic status of *Pisum sativum* L. cv. Cysterski leaves infested by *Acyrthosiphon pisum* Harris, population demographic parameters and *A. pisum* feeding activity. A reduction in the level of semiquinone radicals in pea seedling leaves pretreated with exogenous NO occurred 24 h after *A. pisum* infestation, which was earlier than in non-pretreated leaves. A decrease in the level of O_2_^•−^ was observed in leaves pretreated with GSNO and infested by aphids at 48 and 72 h post-infestation (hpi). Directly after the pretreatment with GSNO, an increase in the level of metal ions was recorded. NO considerably induced the relative mRNA levels for phenylalanine ammonia-lyase in 24-h leaves pretreated with NO donors, both non-infested and infested. NO stimulated the accumulation of pisatin in leaves until 24 h. The Electrical Penetration Graph revealed a reduction in the feeding activity of the pea aphid on leaves pretreated with NO. The present study showed that foliar application of NO donors induced sequentially defense reactions of pea against *A. pisum* and had a deterrent effect on aphid feeding and limited the population growth rate.

## 1. Introduction

Plants, similarly as animals, have an innate immune system which recognizes conserved microbe/pathogens-associated molecular patterns (MAMPs/PAMPs) or herbivore-associated molecular patterns (HAMPs). Although plants do not have mobile defenders or an adaptive immune system, they have their own extensive defense strategies. For example, they can use systemic signals sent from infection/infestation sites [1]. The capacity of plants to mobilize defensive reactions is strongly related to their physiological status [2]. The metabolic status of plants is dependent on the stage of development, nutrition and environmental conditions. In response to attacks of herbivores, they induce a wide spectrum of defense reactions directed against the attackers both at the site of infestation and systemically. Examples of such reactions include global changes in gene expression [3,4], cell wall strengthening [5,6], biosynthesis of secondary metabolites [7,8,9,10] and pathogenesis-related (PR) proteins [11,12]. The above-mentioned plant responses are preceded by the transmission of defense response signal cascades. Signals are mediated by second messengers such as Ca^2+^ ions, protein kinases, reactive oxygen/ nitrogen species (ROS/RNSs) and bioactive endogenous molecules such as phytohormones [13,14]. Therefore, phytophagous insects may encounter a strong defensive barrier as a result of induction of PAMP-triggered immunity (PTI) [15].

The aphid *Acyrthosiphon pisum* Harris is an oligophagous herbivore that infests plants of the Fabaceae family, especially those of the tribes Genistae, Trifoliae, and Fabeae, including plants of high economic importance such as pea *Pisum sativum* L. The feeding of *A. pisum* causes plant injury by removing plant sap with the sucking-piercing mouthparts. Beside direct damage, the pea aphid vectors more than 30 non-persistent and persistent viruses [16].

The present study highlights the effect of known NO donors producing the NO^+^ (sodium nitroprusside, SNP) and NO forms (nitrosoglutathione, GSNO) on demographic parameters of the pea aphid population, feeding activity and defense reactions of pea seedlings. This is the first report revealing the dynamics of defense (changes in the redox status, phenylpropanoid metabolism and β-glucosidase activity) induced by exogenous NO in plant response to aphid attack.

In the past 20 years, the perception of NO has evolved from a gaseous free radical to a molecule that plays a crucial role in diverse physiological processes [17]. The regulatory function of NO is primarily associated with post-translationally modified proteins. NO can control physiological processes also directly by regulating gene transcription [18]. As reported by Corpas and Barroso [19], nitric oxide (NO), peroxynitrite (ONOO^−^) and *S*-nitrosoglutathione (GSNO) are components which have important signaling functions in higher plants under physiological and stress conditions. These molecules can bind to metal centres, contribute to *S*-nitrosylation of thiol groups and nitration of tyrosine [20,21,22,23]. Therefore, NO may directly affect the activity of plant proteins and the signaling cascade leading to changes in gene expression [24,25,26,27]. NO can influence transduction processes by the regulation of key signaling proteins, i.e. protein kinases (MAPK, CDPK and SnRK) and Ca^2+^-permeable channels, as well as the mobilization of secondary messengers including intracellular Ca^2+^, cGMP, and cADPR, and phosphorylation-dependent processes [22,28]. Therefore, the cross-talk interactions between NO, Ca^2+^, and protein kinases in plant cells exposed to environmental stimuli might influence the dynamic of gene expression regulation. In the past 20 years, the perception of NO has evolved from a gaseous free radical to a molecule that plays a crucial role in diverse physiological processes [17]. The regulatory function of NO is primarily associated with post-translationally modified proteins. NO can control physiological processes also directly by regulating gene transcription [18]. As reported by Corpas and Barroso [19], nitric oxide (NO), peroxynitrite (ONOO^−^) and *S*-nitrosoglutathione (GSNO) are components which have important signaling functions in higher plants under physiological and stress conditions. These molecules can bind to metal centres, contribute to S-nitrosylation of thiol groups and nitration of tyrosine [20,21,22,23]. Therefore, NO may directly affect the activity of plant proteins and the signaling cascade leading to changes in gene expression [24,25,26,27]. NO can influence transduction processes by the regulation of key signaling proteins, i.e. protein kinases (MAPK, CDPK and SnRK) and Ca^2+^-permeable channels, as well as the mobilization of secondary messengers including intracellular Ca^2+^, cGMP, and cADPR, and phosphorylation-dependent processes [22,28]. Therefore, the cross-talk interactions between NO, Ca^2+^, and protein kinases in plant cells exposed to environmental stimuli might influence the dynamic of gene expression regulation.

In order to explain the role of NO in the organism commercially available NO donors are used in most experiments [29]. NO localization and release kinetics determine specific functions of this molecule [30]. In plants NO is generated from nitrite (NO_2_^−^), l-arginine and possibly other N-compounds via distinct enzymatic and chemical processes. In nonenzymatic processes NO is released from nitrous acid and *S*-nitrosoglutathione [31]. In in vitro experiments the chemical breakdown of NO donor molecules, i.e. sodium nitroprusside (SNP), *S*-nitroso-*N*-acetylpenicillamine (SNAP), *S*-nitrosoglutathione (GSNO) and 3-morpholinosydnomine(SIN-1), leads to the release of NO [32,33]. The influence of NO donor, *S*-nitrosoglutathione (GSNO) and sodium nitroprusside (SNP) on the plant immune response was demonstrated in studies concerning the impacts of biotic factors such as fungal pathogens [34,35]. In turn, for abiotic stresses the application of an NO donor (e.g., SNP) was shown to reduce detrimental effects of abiotic stressors [36,37]. Studies on NO, being a reactive nitrogen species, have shown its effect on different cells to be either protective or toxic, depending on its concentration and on the location [20]. Moreover, ROS may be scavenged by NO, which is an antioxidant agent as well as a signaling molecule, thus altering antioxidative gene expression and as a result protecting plant cells against oxidative damage [37]. On the other hand, Davies [38] reported that in insects NO signaling is involved in various physiological processes, such as reproduction, locomotion, olfaction, learning and memory [39]. Moreover, research conducted by Liu [40] suggests an insecticidal activity of NO. Being a signal molecule in biological systems nitric oxide proved to be an effective and safe agent applicable in insect control under ultralow oxygen conditions.

The preliminary aim of our studies was to verify the effect of exogenous NO, i.e., *S*-nitrosoglutathione (GSNO) or sodium nitroprusside (SNP), on demographic parameters of *Acyrthosiphon pisum* population and feeding activity. Apart from these investigations the primary objective was to verify if exogenous NO induces defense reactions in leaves of pea seedlings (*Pisum sativum* L. cv. Cysterski) against *A. pisum* Harris. Therefore, initial investigations concerned the effects of NO donors alone and cross-talk of exogenous NO and *A. pisum* infestation on changes in cell redox status, i.e., concentrations of free radicals and levels of Mn^2+^ and Fe^3+^ ions, with the use of electron paramagnetic resonance spectroscopy (EPR). Moreover, one aim of the research was to determine the expression of genes encoding phenylalanine ammonialyase (PAL), as an enzyme initiating phenylpropanoid metabolism, and the levels of flavonoids, including pisatin, in leaves exposed to NO donors, either *A. pisum* infested and non-infested. In addition, changes in β-glucosidase activity, the enzyme hydrolysing free aglycones from their glycoside bonds, were determined. Therefore, in the present study we showed how pretreatment with NO donors influences defense reactions sequence and its intensity in the leaves of pea seedlings. Identification of defense responses of pea leaves in the context of the pretreatment with exogenous NO and the impact of NO on *A. pisum* population growth rate and feeding activity is completely novel. In order to develop long-term and sustainable aphid control strategies it is crucial to understand the role of plant immunity and the induction of defense against aphids.

## 2. Results

### 2.1. The Effect of Nitric Oxide Donors on Demographic Parameters of *Acyrthosiphon pisum* Population

**Figure 1:**
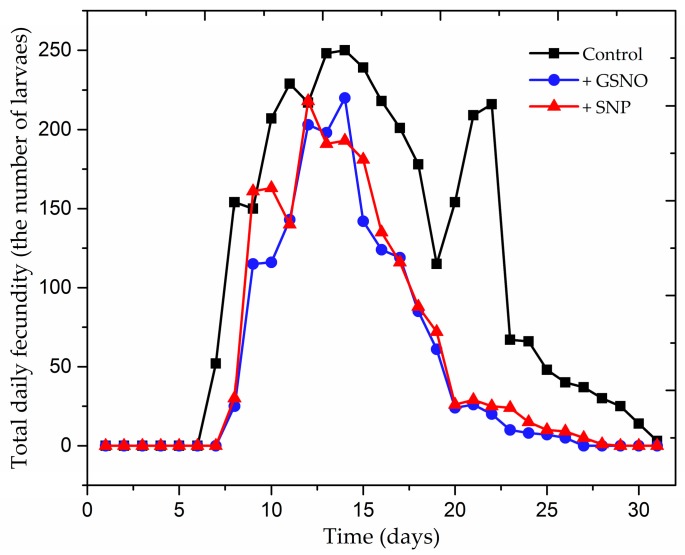
Effect of nitric oxide donors on fecundity of 20 apterous females *Acyrthosiphon pisum* cultured on the *Pisum sativum* L. cv. Cysterski seedlings. Values are means ± SE from 20 replicates per treatment. Hypotheses on equality of means were verified by the two-sample *t*-test (Table S1). To account for multiple testing, we used the Bonferroni correction.

The treatment of pea seedlings with exogenous NO extends the pre-reproductive period by ca. 1 day, but it does not affect the survival of larvae (Table 1). The observations revealed that in the control 100% larvae reached maturity and after GSNO/SNP treatment about 95%. Statistical analysis confirmed significance of differences in these results (Table S1). Also, GSNO and the SNP had a significant impact on the length of the period of reproduction of *A. pisum* (Table 1 and Table 2). Therefore, NO donors limited approximately twice the duration of the period of reproduction and influenced the decrease in fertility of *A. pisum* (Figure 1). The highest rm (intrinsic rate of increase) was recorded for *A. pisum* cultivated on control seedlings (untreated NO donor) (0.270), and the lowest rm was found for aphids cultured on seedlings treated with GSNO (0.217) (Table 2). Analysis of demographic parameters revealed that, within a 24-h period on the control seedlings, the population of *A. pisum* increases 1.31 times, whereas on seedlings treated with GSNO, the population of *A. pisum* increased 1.242 times; whereas on seedlings treated with SNP, it increased 1.257 times. Mean generation time (T) of *A. pisum* cultured on seedlings from all experimental variants (control and GSNO/SNP) was approximately 13 days and at that time the aphid population was increasing 33.672 times for the control, for aphids cultured on seedlings treated with SNP it was 18.22 times and for aphids cultured on seedlings treated with GSNO 16.510 times.

### 2.2. Effect of Exogenous Nitric Oxide on Behavioral Responses of *A. pisum* during Probing in Plant Tissues of Pea

The analysis of EPG recordings exposed various aphid behaviors associated with stylet position both inside and outside plant tissues: non-penetration (np), probing in the mesophyll (C) including the so-called “derailed” stylet movements (F), probing in phloem tissue including salivation (E1) and sap ingestion (E2), and ingestion of xylem sap (G). “Derailed” stylet movements (F) and the xylem phase (G) occurred sporadically irrespective of treatment, therefore they were analysed together with pathway activity (C) and defined as probing in non-phloem tissues. The typical behavior of *A. pisum* on the control seedlings consisted mainly of probing activities (>90% of experimental time) that were associated with the pathway and phloem phases (phloem phase index: 0.6). Probing was divided into 54 probes on average and 10% of these probes contained a phloem phase. On average, the time to reach phloem sieve elements was 2 h and the delay between finding sieve elements (1st period of phloem salivation E1) and sustained sap uptake (E2 longer than 10 min) was 0.1 h. There were 10.4 phloem phase periods per aphid. These phloem phases included primarily sap consumption: the proportion of saliva secretion in aphid activity in sieve elements was very low (phloem salivation index: 0.02) (Table 3). Aphid probing behavior on 24 h +GSNO seedlings did not differ significantly from individuals on control seedlings: the duration, frequency and sequence of individual phases of probing, including the phloem phase, were similar. In contrast, significant changes in the pea aphid probing activities occurred on 48 h +GSNO plants in relation to the control and 24 h +GSNO seedlings: the occurrences of the first phloem phase E1 and the first sustained ingestion phase E2 > 10 min. were delayed by approximately five and four hours, respectively, and the share of the phloem phase in probing activities decreased by 20% (Table 3). On 24 h +SNP and 48 h +SNP seedlings there occurred an increasing tendency for aphids to keep stylets outside plant tissues (i.e., longer non-probing periods and shorter pathway activity). The time dedicated to activities during probing in sieve elements was comparable to the duration of these activities on control plants, but aphids on 24 h +SNP seedlings reached phloem vessels and started sap ingestion significantly later than on control seedlings. Similar tendencies were found for aphids on 48 h +SNP seedlings. No statistically significant differences were observed in behavior between aphids on 24 h +SNP and 48 h +SNP seedlings (Table 4).

### 2.3. The Effect of Nitric Oxide Donors and *A. pisum* on Semiquinone Radical Generation

**Figure 2:**
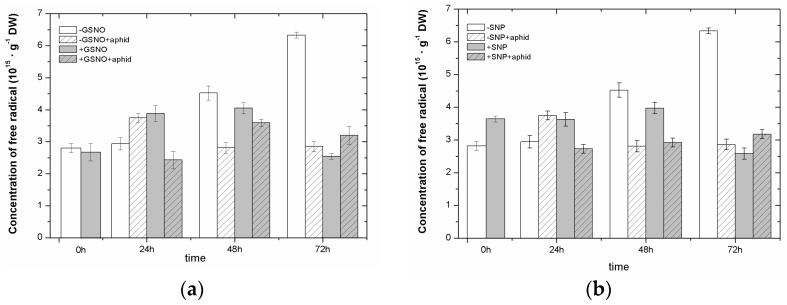

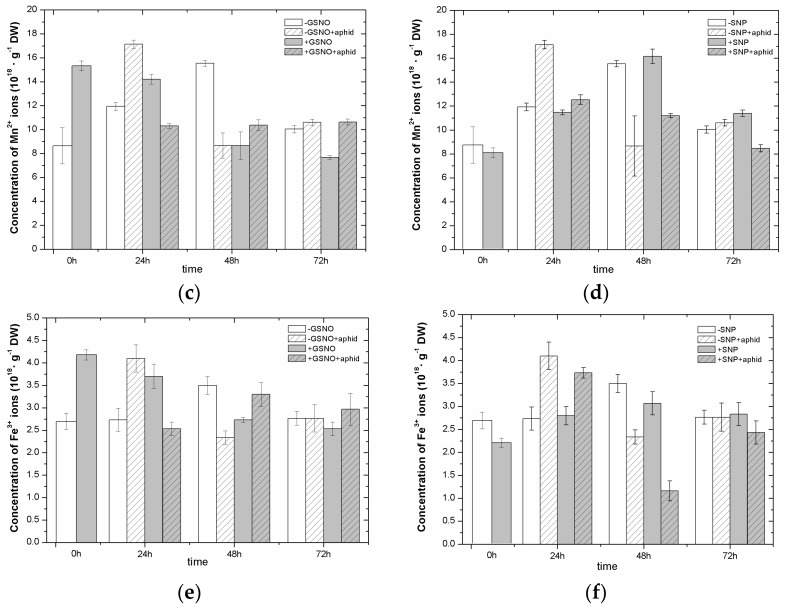
Effect of nitric oxide donors and *A. pisum* on concentrations of: semiquinone radical (**a**,**b**); manganese (**c**,**d**); and iron ions (**e**,**f**) in leaves of *P. satium* L. cv. Cysterski . The data were obtained in three independent experiments and statistically analysed using ANOVA (*p*-values at α = 0.05). Hypotheses on the equality of means were verified by the two-sample *t*-test. To account for multiple testing, we used the Bonferroni correction (statistically significant differences are shown in Table S2).

*A. pisum* feeding alone caused an increase in the concentration of semiquinone radicals with one *g*-values, 2.0026 ± 0.0005 in leaves, but it was observed only at 24 hpi. In turn, in subsequent time points after *A. pisum* feeding in −GSNO + aphid/−SNP + aphid leaves concentrations of these radicals decreased significantly in relation to the control leaves (−GSNO/−SNP). Pretreatment with SNP, but not GSNO, stimulated generation of semiquinone radicals already at 0 h of experiment (24 h after application), which was detected using electron paramagnetic resonance spectroscopy (EPR) (Figure 2a,b); the concentration of these radicals in pea leaves pretreated with SNP (+SNP) was 29% higher than in non-pretreated leaves (−SNP). Furthermore, already at 24 hpi and then at 48 hpi in leaves pretreated with GSNO/SNP and infested by *A. pisum* (GSNO + aphid/SNP + aphid), a reduction in semiquinone radicals in relation to pretreated and non-infested (+GSNO/+SNP) leaves was recorded. Statistical analysis showed highly significant differences in these results (Table S2).

### 2.4. The Effect of Nitric Oxide Donors and *A. pisum* on the Concentration of Manganese (Mn^2+^) and Iron (Fe^3+^) Ions

EPR spectroscopy revealed the presence of Mn^2+^ ions with *g*-values of 2.00 (0.01) (Figure 2c,d) and Fe^3+^ ions with *g*-values of 4.27 (Figure 2e,f) in leaves. Level of these ions decreased in leaves infested by *A. pisum* and non-pretreated with NO donors (−GSNO + aphids/−SNP + aphids) (by approximately 62% and 33%), but it was only at 48 hpi. In turn, GSNO foliar application resulted in a considerable increase in the concentrations of Mn^2+^ and Fe^3+^ ions in +GSNO leaves in relation to the control (−GSNO) at 0 h of experiment (Figure 2c,e). Statistical analysis showed differences in the above-mentioned results to be highly significant (Table S2). In the case of SNP, an increase in the concentration of these ions was observed from 0 to 48 h of experiment in +SNP leaves as compared to −SNP leaves (Figure 2d,f). However, the highest accumulation of these ions was found in 24-h leaves non-pretreated with NO donors and leaves infested by *A. pisum* (−GSNO + aphids/−SNP + aphids). An opposite trend was observed in 24-h leaves pretreated with the GSNO donor and infested by *A. pisum* (+GSNO + aphids), wherelevels of Mn^2+^ and Fe^3+^ ions decreased (Figure 2c,e). Reduced levels of these ions were recorded at 48 and 72-h in leaves pretreated with SNP and infested by aphids (+SNP + aphids) in relation to pretreated with SNP and non-infested (+SNP) leaves (Figure 2d,f). Therefore, at 48 and 72 hpi, the concentration of Mn^2+^ ions in −SNP leaves was by approximately 31% and 22% lower than in +SNP leaves, respectively.

### 2.5. The Effect of Nitric Oxide Donors and *A. pisum* on Superoxide Anion Generation

**Figure 3:**
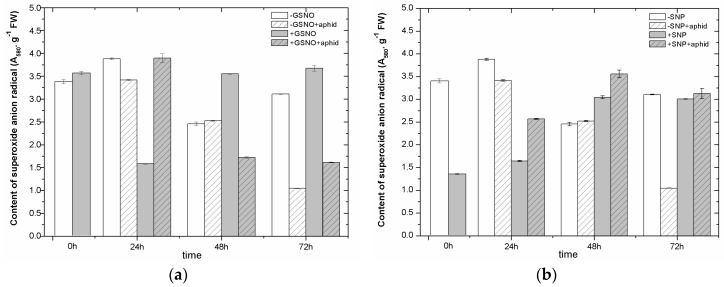
The effect of nitric oxide donors (GSNO (**a**); and SNP, (**b**)) and *A. pisum* on the generation of the superoxide anion in leaves of *P. satium* L. cv. Cysterski. The data were obtained in three independent experiments and statistically analysed using ANOVA (*p*-values at α = 0.05). Hypotheses on the equality of means were verified by the two-sample *t*-test (Table S2). To account for multiple testing, we used the Bonferroni correction.

At 0 h of experiment, the reduction in the level of superoxide anion by approximately 61% was noted in leaves pretreated with SNP (+SNP) in relation to non-pretreated (−SNP), with the differences in the results being statistically significant (Figure 3b) (Table S2). Such a reduction was not found in the case of leaves pretreated with GSNO (+GSNO) at 0 h of experiment, but at 24 h of experiment (+GSNO vs. −GSNO) (Figure 3a). At the subsequent time points, the level of O_2_^•−^ remained high in leaves pretreated with GSNO (+GSNO). In turn, *A. pisum* infestation caused high generation of O_2_^•−^ in +GSNO + aphid leaves at 24 hpi, followed by a considerable decrease in its level (more than 50%) in relation to +GSNO leaves at 48 and 72 hpi. Moreover, *A. pisum* feeding, particularly at 72 hpi, caused a very strong reduction in O_2_^•−^ in −GSNO + aphids leaves. It must be stressed that the impact of SNP, i.e., the NO^+^ donor, on the generation of O_2_^•−^ in leaves infested by *A. pisum* was different than GSNO, the NO·donor, because in 48 and 72-h +SNP + aphids leaf tissues, the level of O_2_^•−^ was exceeded that in +SNP leaf tissues. The highest generation of O_2_^•−^ in these tissues was noted in 48-h leaves pretreated with SNP and infested by *A. pisum*.

### 2.6. The Effect of Nitric Oxide Donors and *A. pisum* on Expression Levels of Phenylalanine Ammonialyase Genes

**Figure 4:**
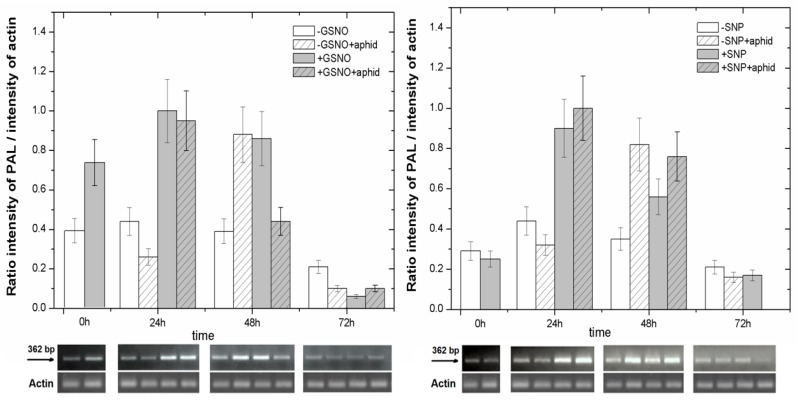
RT-PCR products amplified from the PAL genes obtained from pea seedling leaves pretreated with NO donors (GSNO or SNP) or non-pretreated, both in the non-infested and infested by *A. pisum*, constitutive actin expression and a ratio of intensity of PAL/intensity of actin. The data were obtained in three independent experiments and statistically analysed using ANOVA (*p*-values at α < 0.05). Hypotheses on the equality of means were verified by the two-sample *t*-test (Table S2). To account for multiple testing, we used the Bonferroni correction.

Semi-quantitative reverse transcription-polymerase chain reaction (RT-PCR) analysis revealed that pea feeding by itself strongly stimulated PAL gene expression at 48 hpi; relative mRNA levels recorded for these genes in −GSNO + aphid/−SNP + aphid leaves were significantly higher than in −GSNO/−SNP leaves. Moreover, already at the start of the experiment (0 h, i.e., 24 h from the moment of pretreatment with GSNO), an increase in the expression of genes encoding phenylalanine ammonialyase (PAL) increased (Figure 4). Then, at 24 and 48 h both GSNO and SNP significantly induced relative mRNA levels of these genes in leaves pretreated with NO donors; the expression level of PAL in pretreated leaves was higher than in non-pretreated (+GSNO/+SNP vs. −GSNO/−SNP). Statistical analysis showed highly significant differences in these results (Table S2). The highest relative mRNA levels for PAL was observed in 24-h leaves pretreated with NO donors, irrespective of A. pisum infestation. In turn, at 72 h the mRNA level considerably decreased in all experimental variants.

### 2.7. The Effect of Nitric Oxide Donors and *A. pisum* on Accumulation of Pisatin

**Figure 5:**
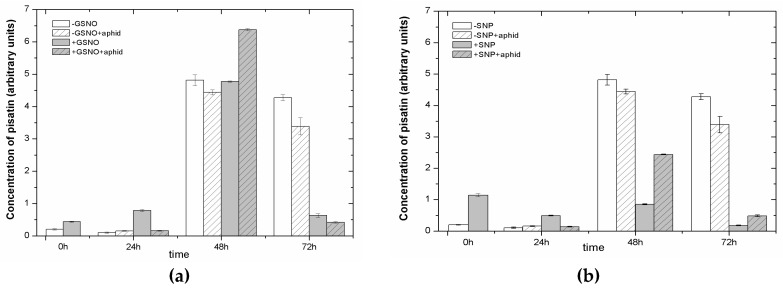
The effect of nitric oxide donors and *A. pisum* on pterocarpan pisatin levels in seedling leaves of *Pisum sativum* L. cv. Cysterski. The data were obtained in three independent experiments and statistically analysed using ANOVA (*p*-values at α = 0.05). Hypotheses on the equality of means were verified by the two-sample *t*-test (Table S2). To account for multiple testing, we used the Bonferroni correction.

The colonization of leaves by *A. pisum* alone did not cause pisatin accumulation in this experimental system. Foliar application of NO donors induced pisatin accumulation in +GSNO/+SNP leaves at 0 and 24 h of the experiment (Figure 5). Therefore, two- and six-fold increase in pisatin levels was found in 0-h leaf tissue of +GSNO/+SNP (24 h after pretreatment with NO) as compared to leaf tissue of −GSNO/−SNP, respectively. Statistical analysis showed highly significant differences in these results (Table S2). In turn, at 24-h, for +GSNO/+SNP leaves, this level was by approx 16% and 23% higher than in non-pretreated, i.e., −GSNO/−SNP leaves. However, the highest accumulation of pisatin was noted at 48 hpi in leaves pretreated with GSNO and infested by *A. pisum* (+GSNO + aphids). In the case of SNP application, at 48 hpi, an approximately three-fold higher level of pisatin was observed in +SNP + aphids leaves than in +SNP leaves, but it was lower from that in other experimental variants (−SNP and −SNP + aphids). Moreover, when analyzing the level of pisatin versus time, it can be observed that from 24 h of experiment the level of this isoflavonoid considerably increased in all experimental variants.

### 2.8. The Effect of Nitric Oxide Donors and *A. pisum* on the Level of Isoflavonoid and Flavonoid Glycosides

**Figure 6:**
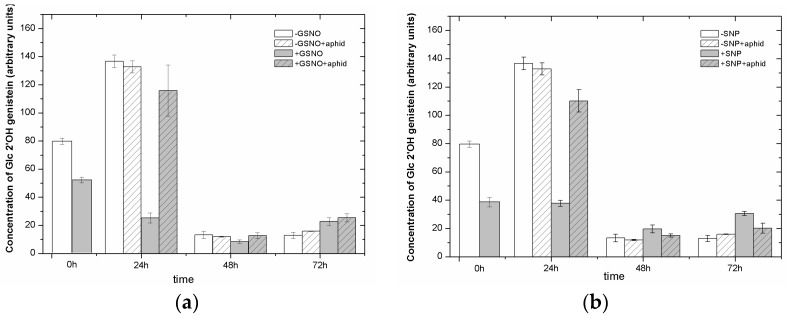

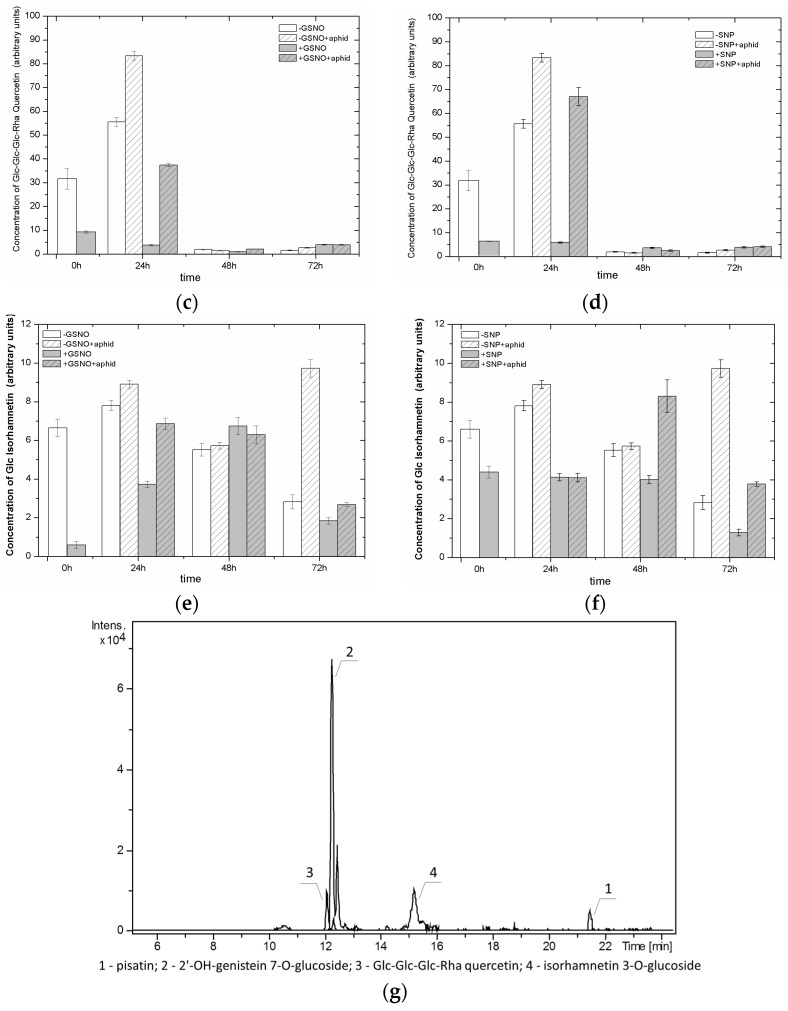
The influence of nitric oxide donors and *A. pisum* on the level of isoflavonoid and flavonoid glycosides: 2′-OH-genistein 7-O-glucoside (**a**,**b**); and quercetin rhamnosyl-triglucoside (**c**,**d**); and isorhamnetin 3-O-glucoside (**e**,**f**). An LC-MS extracted ion chromatogram showing profiles of phenolic compounds found in pea leaves (**g**). The data were obtained in three independent experiments and statistically analysed using ANOVA (*p*-values at α = 0.05). Hypotheses on equality of means were verified by the two-sample *t*-test (Table S2). To account for multiple testing, we used the Bonferroni correction.

At 0 h and 24 h of experiment, a 2- and 3.5-fold decrease in the level of isoflavone glycoside 2′-OH-genistein 7-*O*-glucoside was noted in leaves pretreated with GSNO/SNP (+GSNO/+SNP) in relation to non-pretreated leaves (−GSNO/−SNP), respectively (Figure 6a,b). In turn, in 24-h leaves pretreated with NO donors and infested by *A. pisum*, approximately 5- and 3-fold higher levels of 2′-OH-genistein 7-*O*-glucoside in +GSNO + aphid/+SNP + aphid leaves than in +GSNO/+SNP leaves was visible, respectively. In the next time points of experiment, the levels of this glycoside declined drastically in all leaf tissues in relation to previous times. Moreover, at 48 and 72 hpi, slightly lower levels of 2′-OH-genistein 7-*O*-glucoside were observed in leaves pretreated with SNP and infested by *A. pisum* (+SNP + aphids) than in +SNP leaves. Liquid chromatography–mass spectrometry analyses revealed the presence of quercetin rhamnosyl-triglucoside (Glc-Glc-Glc-Rha quercetin) (Figure 6c,d,g) and isorhamnetin 3-*O*-glucoside (Figure 6e–g), which are flavonol glycosides. At 0 h of experiment, similarly to 2′-OH-genistein 7-*O*-glucoside, the level of quercetin rhamnosyl-triglucoside (Glc-Glc-Glc-Rha quercetin) decreased in leaves after the pretreatment with NO donors (24 h after application) (Figure 6c,d). In 24-h leaves pretreated and non-pretreated with exogenous NO, non-infested (+GSNO)/+SNP or −GSNO/−SNP) and infested (+GSNO + aphid/+SNP + aphid or −GSNO + aphid/−SNP + aphid), the levels of this metabolite demonstrated the same trend as 2′-OH-genistein 7-*O*-glucoside. Therefore, *A. pisum* infestation induced a high accumulation of quercetin rhamnosyl-triglucoside in leaves pretreated and non-pretreated with exogenous NO, but the level of this glucoside was higher in non-pretreated and infested leaves (−GSNO + aphid/−SNP + aphid) than in pretreated and infested leaves (+GSNO + aphid/+SNP + aphid). From 24 h of experiment the levels of this glycoside showed a drastic decline in all experimental variants. Generally at all-time points of the experiment, the level of isorhamnetin 3-*O*-glucoside in leaves pretreated with GSNO/SNP was lower than in non-pretreated leaves (Figure 6e,f). *A. pisum* infestation significantly stimulated accumulation of isorhamnetin 3-glucoside, but higher levels of this glucoside were recorded in −GSNO + aphid/−SNP + aphid leaves than in +GSNO + aphids/+SNP + aphids leaves. The highest levels of this metabolite was found in 72-h −GSNO + aphids/−SNP + aphids leaves. Therefore, in leaves pretreated with GSNO/SNP and infested by the pea aphid (+GSNO + aphid/+SNP + aphid), levels of isorhamnetin 3-*O*-glucoside were 2.68–6.86 arbitrary units (Figure 6e) and 3.76–8.31 arbitrary units (Figure 6f), respectively, while the levels in leaves non-pretreated with GSNO/SNP and infested by the pea aphid (−GSNO + aphid/−SNP + aphid) ranged from 5.73 to 9.73 arbitrary units.

### 2.9. The Effect of Nitric Oxide Donors and *A. pisum* on β-Glucosidase Activity

**Figure 7:**
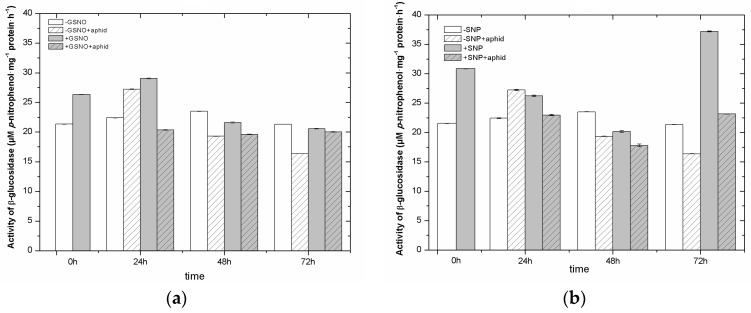
The effect of nitric oxide donors (GSNO (**a**); and SNP (**b**)) and *A. pisum* on β-glucosidase activity. The data were obtained in three independent experiments and statistically analysed using ANOVA (*p*-values at α = 0.05). Hypotheses on equality of means were verified by the two-sample *t*-test (Table S2). To account for multiple testing, we used the Bonferroni correction.

It has been demonstrated that NO donors alone stimulated the activity of β-glucosidase already at 0 h (Figure 7). Statistical analysis showed highly significant differences in these results (Table S2). Also, in 24-h leaves pretreated with GSNO/SNP, the activity of β-glucosidase was higher than in non-pretreated and non-infested leaves (−GSNO/−SNP). Particularly the high activity of the enzyme was noted in 72-h leaves pretreated with SNP (+SNP) and the activity of this enzyme was at least 1.5 times higher than in other variants (–SNP, –SNP + aphid and +SNP + aphid) (Figure 7b). Besides, pea aphid feeding alone (−GSNO + aphid/−SNP + aphid) caused an increase in β-glucosidase activity, but only at 24 hpi in relation to non-pretreated and non-infested leaves (−GSNO/−SNP). Moreover, it is surprising that the activity of β-glucosidase in leaves infested by *A. pisum*, both pretreated with GSNO/SNP (+GSNO + aphid/+SNP + aphid) and non-pretreated (−GSNO + aphid/−SNP + aphid), was lower than in non-infested leaves (+GSNO/+SNP and −GSNO/−SNP).

## 3. Discussion

This study is the first to demonstrate that application of NO (GSNO or SNP) on pea seedlings reduce *A. pisum* performance and feeding activity. At the same time, we showed here the dynamics of defense (e.g., changes in the redox status, phenylpropanoid metabolism and β-glucosidase activity) induced by exogenous NO in *P. sativum* response to pea aphid attack. Therefore, analysis of demographic parameters of the population of *A. pisum* revealed that NO donors prolong the pre-reproductive period, had a significant impact on the length of the reproduction period of *A. pisum* reducing it approximately two-fold (Table 1 and Table 2). Additionally, it has been demonstrated that GSNO and SNP have a significant impact on reducing fertility (Table 1 and Table 2, Figure 1). Thereby, this study highlights the role of NO donors in the limiting of the *A. pisum* population growth rate. It has been reported that NO may be an environmentally friendly option for postharvest pest control. For example, Liu [40] reported efficacy of NO fumigations under ultralow oxygen levels in relation to all life stages of the four analysed insect species, i.e., western flower thrips, *Frankliniella occidentalis* (Pergande); aphid, *Nasonovia ribisnigri* (Mosley); confused flour beetle, *Tribolium confusum* Jacquelin du Val; and rice weevil, *Sitophilus oryzae* (L.). Besides, it is important to remember that the efficacy of NO fumigation is higher than that of phosphine and comparable with that of methyl bromide, while at the same time being safer to human health and quality of fresh produce. On the other hand, as reported Ascenzi and Gradoni [41], NO can limit *Trypanosoma*, *Plasmodium*, and *Schistosoma* development at all stages of the parasite life cycle. Strong evidence has been provided by basic studies in comparative physiology and biochemistry in simple organisms that NO signaling is evolutionarily and functionally conserved [38]. NO has been used as a signaling molecule in all invertebrate orders investigated to date [42]. Therefore, salivary NO is used as a vasodilator by blood-sucking insects [43] and l-glutamate-stimulated NO signaling was discovered in *Trypanosoma cruzi* epimastigotes [44]. In turn, in *Hydra vulgaris*, NO signaling contributed to olfactory-like feeding behavior [45] and a calcium-sensitive NOS was implicated in *Sepia officinalis* in melanin production in their ink glands [46]. Additionally, the neuronal role of NO signaling was investigated by Scholz et al. [47]. It has been demonstrated that in lobsters the NO/cGMP signaling pathway may be involved in the development of the nervous system. Furthermore, NO may be a modulatory neurotransmitter for various neurons throughout the central nervous system (CNS).

Results obtained within these studies provided also an answer to the important question: to what degree NO in leaves of pea seedlings influence the intensity of feeding by *A. pisum* that possesses piercing-sucking mouthparts. The application of NO donors GSNO and SNP to pea seedlings caused similar significant changes in pea aphid feeding activity during the pre-phloem and the phloem phases, which were the delay in reaching phloem vessels they had access to plants, as well as a decrease in phloem phase duration, respectively (Table 3 and Table 4). However, the timing and occurrence of alterations in probing activities differed depending on the donor and the time interval between the application and aphid access. The application of SNP evoked changes in aphid activities sooner than GSNO: the delay in reaching sieve elements and the reduced phloem sap consumption occurred in aphids on plants that were offered as soon as 24 h after treatment while on GSNO-treated plants only after 48 h. In turn, other studies have revealed that the delay in reaching sieve elements can be associated with the occurrence of xenobiotics in non-phloem tissues, especially the mesophyll: on the way from the epidermis to the phloem, aphids puncture mesophyll cells with their stylets and ingest samples of cell contents for gustatory purposes, which is supposed to help in the recognition of the host plants under natural conditions [48,49,50]. Under experimental conditions, the delay or a failure in reaching phloem vessels was often recorded following the exogenous application of chemical deterrents [51,52]. Likewise, the reduction of time assigned to the uptake of phloem sap indicates the activity of deterrent factors located in sieve elements [53,54,55]. On acceptable plants sap consumption may last for several hours [56,57].

In parallel, we investigated the potential defense mechanisms of plant-host such as changes in the level of free radicals, PAL gene expression, flavonoids and β-glucosidase activity. These changes in metabolic status of *P. sativum* L. seedlings stimulated by NO may contribute to reducing the feeding activity of *A. pisum*. Our understanding of the role played by NO in plant defense against aphids is still fragmentary and strongly limited. As reported by Bellin et al. [58], NO is an essential component of plant immune response. It is known that the quickness and the rate of startup responses decide about the success of a given defensive strategy of the plant against biotic factors. However, little is known on the time-dependent aspect of changes induced by NO donors (GSNO and SNP) and cross-talks between NO and *A. pisum* infestation. Molecular and metabolome changes were studied at 0 and 24, 48 and 72 h after *A. pisum* infestation.

The results of this study show that free radicals (semiquinone radicals), which gave signals with one *g*-values, 2.0026 ± 0.0005 (the line width 7.5 Gs on the X-band and Q-band) were found in the leaves pretreated with exogenous NO or non-pretreated as well as in non-infested or infested by *A. pisum* (Figure 2a,b). Additionally, electron paramagnetic resonance (EPR) spectroscopy revealed also signals of Mn^2+^ ions with *g*-values of 2.00(0.01) (Figure 2c,d) and Fe^3+^ ions with *g*-values of 4.27 (Figure 2e,f) in the above types of these leaves. The reduction in semiquinone radical levels in pea seedling leaves pretreated with exogenous NO, already 24 h after *A. pisum* infestation and later (48 hpi), may suggest that semiquinone radicals may be incorporated in polymers, e.g., lignin, in combination with other reactive free radicals (e.g., O_2_^•−^), which propagate depolymerization throughout the lignin matrix and prevent the breakdown of cell walls. It was demonstrated by Pearce et al. [59] that semiquinone radicals can interact as free radical scavengers, quenching free radical chain reactions leading to lignocellulose biodegradation. Our research demonstrated that already at 24 hpi the reduction in semiquinone radicals in 24-leaves pretreated with exogenous NO (+GSNO + aphid/+SNP + aphid) was accompanied by a high level of O_2_^•−^ (Figure 3). Perhaps, the maintaining of a high level of O_2_^•−^ generation is necessary at 24 hpi, because a pool of O_2_^•−^ reacts with semiquinone radicals, and a portion of O_2_^•−^ is needed for cytotoxic effects against *A. pisum*. The wide-ranging impact of NO on the metabolism of plants is associated with two of its functions, i.e., cytoprotective and cytotoxic, in which NO may play in the interaction with ROS. Bellin et al. [58] reported that during defense ROS function either independently or in cooperation with NO, modulating the RNS signaling functions throughout the process. Reduction in the level of semiquinone radicals noted in the present study may indicate the induction of a defensive barrier by NO already in the early stages of *A. pisum* feeding. In leaves non-pretreated with NO donors the same trend for the reduction in semiquinone radicals also was noted in response to *A. pisum* infestation as in pretreated with NO, but it occurred later. Substantiated experimental evidence clearly shows that NO being a free radical molecule acts as a mediator in biochemical processes related to a broad spectrum of physiological events in plants [60]. In the response of plants to aphids, mechanisms of intracellular redox regulation of metabolic processes are crucial. In addition, in the present study it is worth mentioning that already at 0 h the elevated generation of semiquinone radicals was observed in +SNP leaves. SNP-induced generation of semiquinone radicals can have a toxic effect towards the pea aphid. Also, our previous experiments in another experimental system revealed the participation of semiquinone radicals in response to *A. pisum* [61]. It has been revealed that aphid infestation enhanced semiquinone radical generation and the superoxide anion in leaves of pea seedlings cultured in perlite and infested by *A. pisum* populations of various sizes, i.e., 10, 20 and 30 aphids.

Plant responses to insects are initiated at the plant cell plasma membrane, where insect herbivores interact both physically (causing mechanical damage) and chemically (introducing elicitors or triggering plant-derived signaling molecules) [62]. Both results from GSNO + aphid (Figure 3a) and SNP + aphid variants (Figure 3b) show different trend for post-infestation O_2_^•−^ generation at 48 and 72 hpi. Firstly, GSNO and *A. pisum* infestation caused a strong decrease in O_2_^•−^ in relation to +GSNO variants at these time points after infestation. In turn, in the case of impact of SNP and *A. pisum* infestation, a higher level of superoxide anion in relation to SNP variant was found at the above-time points after infestation. Sometimes the differences in the impact of NO donors may result from the fact that from the GSNO, NO is formed and from SNP—the NO^+^ is formed. NO burst and hypothetical modes of NO action in plant defense responses are connected with the activation of a signal transduction cascade, including an oxidative burst and resulting in the production of ROS [63]. As reported by Bolwell and Wojtaszek [64], the formation of ROS is part of the antimicrobial response in plants. Moreover, Moloi and Westhuizen [65] studies suggested that peroxynitrite can be involved in resistance responses to Russian wheat aphid (RWA) as one of the signal molecules in addition to NO, H_2_O_2_ and SA. Besides, these authors revealed the significant effect of NO application (using SNP donor) on the reduction of the RWA population growth and the feeding symptoms. On the other hand, Foisssner et al. [66] reported that ROS and NO interact to execute pathogens by forming a highly toxic peroxynitrite (ONOO^−^). Intra- or extracellular NO might act as a long distance, mobile signal triggering the development of systemic resistance. Moreover, in the present study we also revealed that the perception of *A. pisum* infestation by *P. sativum* leaves induces a strong accumulation of Mn^2+^ and Fe^3+^ ions (Figure 2c,d,e,f) already at 0 h of experiment in leaves pretreated with GSNO (+GSNO variant), suggesting their uptake from the medium used in pea seedling culture and their inclusion in the antioxidant defense.

Moreover, the LC-UV/ESI-MS/MS analyses revealed the sequence of changes in the accumulation of secondary metabolites e.g., flavonoids, which may have a deterrent impact on *A. pisum* feeding activity after the application of NO donors pea seedlings (Figure 5 and Figure 6). The accumulation of pisatin, phytoalexin in pea seedling leaves pretreated with GSNO/SNP especially until 24 h was demonstrated (Figure 5a,b and Figure 6g). Also, it should be stressed that GSNO and SNP played a considerable role inducing the expression level of genes encoding PAL, the enzyme initiating phenylpropanoid metabolism (Figure 4). This induction of PAL genes was visible already in 0-h leaves pretreated with GSNO (24 h after application). In turn, the highest accumulation of pisatin was noted at 48 hpi in leaves pretreated with GSNO and infested by *A. pisum* (+GSNO + aphids). Besides, through the amplification of the signal generated by NO and NO and *A. pisum* feeding cross-talks, high mRNA transcript levels for PAL in +GSNO/+SNP and +GSNO + aphid/+SNP + aphid leaves was observed. High levels of mRNA transcripts for PAL were accompanied also by an elevated level of pisatin in 0-h leaves pretreated with GSNO. Moreover, *A. pisum* infestation alone caused increased expression of genes encoding PAL. Based on the results on pisatin and expression genes encoding PAL, it could be concluded that NO itself and NO and *A. pisum* infestation induced the biosynthesis of this phytoalexin. These metabolites are low molecular weight antimicrobial substances produced by plants in response to infection or stress, and involved in their active defense mechanisms [8,67]. Additionally, Morkunas et al. [10] revealed a considerable post-infestation accumulation of pisatin in leaf cells after 48 h of aphid colonization and this increase was correlated with the number of aphids colonizing seedlings. NO is a multi-functional signaling molecule, which modifies various physiological processes in plants [68]. Besides, NO is a short-lived free radical gas, typically applied to plants using NO-releasing compounds such as SNP [69]. Hao [70] showed that NO application via the SNP enhanced UV-B induced PAL activity and increased flavonoid accumulation in *Ginkgo biloba* callus. Moreover, as revealed by Wang and Wu [71], the NO donor SNP alone significantly stimulated PAL activity, while also enhancing methyl jasmonate-induced PAL activity (MeJA +SNP vs. MeJA). In turn, Zhang et al. [72] reported that pretreatment of the cells with SNP increased SA-induced NO generation, PAL activation and salvianolic acid B (Sal B) accumulation, which suggested that NO activated PAL and was involved in SA-induced Sal B biosynthesis. The increase in levels of anthocyanins, ascorbic acid and phenolic compounds triggered by SNP caused a greater antioxidant capacity in SNP treated fruits [73]. Also, earlier studies by Delledonne et al. [74] and Durner et al. [75] showed both that while NO acted synergistically with ROS to potentiate cell death and that it also acted independently of ROS, inducing the expression of defense-related genes, including the PAL gene. In studies presented here, a decline in the level of isoflavone (Figure 6a,b) and flavonol glycosides (Figure 6c–f) in leaves pretreated with NO donors may suggest the need for the formation of free aglycones in these leaves. A very strong reduction in these glucosides was already observed in 0-h tissues pretreated with GSNP/SNP (+GSNO/+SNP variant). Also, it has been revealed that NO donors alone stimulated the activity of β-glucosidase already in 0-h tissues and 24-h tissues (+GSNO/+SNP variant) (Figure 7). What is more, as a consequence of pea aphid feeding β-glucosidase activity increased in 24-h tissues infested by aphids (−GSNO + aphid/−SNP + aphid variants) in relation to non-infested tissues (−GSNO/−SNP variants). Additionally, the activity of β-glucosidase in leaves infested by *A. pisum*, both pretreated with NO donors (+GSNO + aphid/+SNP + aphid) and non-pretreated (−GSNO + aphid/−SNP + aphid) was lower than in non-infested leaves (+GSNO/+SNP and −GSNO/−SNP). In these studies, β-glucosidase activity was measured with a substrate 4-nitrophenyl-beta-D-glucopyranoside, but not with substrate, such as one of the isoflavone glycosides (2′-OH-genistein 7-O-glucoside) and flavonol glycosides (quercetin rhamnosyl-triglucoside) found in the study. As reported by Miller [76], many molecules involved in plant defense, e.g., phenols, isoflavanoids, salicylic acid (SA) and cyanogenic compounds, are released from the glucosylated storage form by β-glucosidases. However, insect adaptations often circumvent or counteract the activity of plant β-glucosidases, the bioactivating enzymes which are crucial in the plant two-component chemical defence [77].

## 4. Materials and Methods

### 4.1. Plant Material and Growth Conditions

Experiments were conducted using pea (*Pisum sativum* L. cv. Cysterski) seeds of the S-elite class, obtained from the Plant Breeding Company at Tulce near Poznan in Poland. Seeds were surface-sterilized as described by Mai et al. [14]. After 6 h of imbibition they were transferred onto filter paper (in Petri dishes) and immersed in a small volume of water to support further absorption. After further 42 h seed coats were removed from the germinating seeds. Next, the germinating seeds (35 pieces) were transferred to hydroponic boxes containing Hoagland medium. Hydroponic boxes were covered with dark foil. Pea seedlings were maintained in a growth chamber at 22–23 °C, 65% relative humidity, and light intensity of 130–150 μM photons m^−2^·s^−1^ with the 14/10 h (light/dark) photoperiod. Hoagland medium was aerated each day using the aeration system.

Experiments were conducted on leaves of 12-day-old pea seedlings subjected to pretreatment with a specific NO donor, i.e., 200 μM GSNO/SNP, or not pretreated with GSNO/SNP (pretreated with H_2_O) in the light at 25 °C. Therefore, the whole pea seedlings growing on the hydroponic boxes containing Hoagland medium in glass boxes were thoroughly sprayed with a solution of GSNO/SNP or H_2_O in the light. Upon light irradiation SNP releases nitric oxide in the form of nitrosonium cation (NO^+^). Twenty-four hours after pretreatment of leaves with GSNO/SNP or with H_2_O (time 0 hpi) they were either infested with *A. pisum* (+GSNO + aphid/+SNP + aphid or −GSNO + aphid/−SNP + aphid), or remained non-infested (+GSNO)/+SNP or −GSNO/−SNP). Because from a strictly chemical point of view, SNP is a NO^+^ donor, the effect of another NO donor, i.e., *S*-nitrosoglutathione (GSNO), we also tested. Leaves of pea seedlings were carefully collected at 0, 24, 48, 72 h post-infestation (hpi) with pea aphids.

### 4.2. Aphids and Experiment on Infestation

*Acyrthosiphon pisum* (Harris), originally kept and supplied by the Department of Entomology, the Poznań University of Life Sciences, Poland, was reared on *Pisum sativum* L. cv. Cysterski in a growth chamber under conditions specified above on the 12th day of culture, each pea seedling was separated and infested with 20 apterous adult females of *A. pisum*. The aphid populations were monitored daily throughout all experiments and newborn nymphs were removed as they appeared. The control plants were pea seedlings with no pea aphid infestation.

### 4.3. Determination of the Effect of Nitric Oxide Donors on the Demographic Parameters of *A. pisum* Population

The whole 12-day-old pea seedlings growing on the hydroponic boxes containing Hoagland medium in glass boxes were thoroughly sprayed with a solution of 200 μM GSNO/SNP or H_2_O (control) in the light. Next, for the influence of GSNO/SNP on the population growth rate, 30 females (apterous aphids of the same age) were individually placed on pea seedling shoots, 24 h after pretreatment of seedlings with a specific NO donor. Larvae born by 30 females were the material for further observations. The survival rate of larvae was determined for a population of 100 larvae. For the calculation of demographic parameters of populations, 20 females were observed. The length of the following developmental stages was recorded: pre-reproduction, reproduction, postreproduction, the total life span and female fecundity. The observations were carried out seven times a week. Demographic parameters for the aphid populations at different variant (control, +GSNO, +SNP) were determined following methods described by Birch [78]:

(a) intrinsic rate of increase, *r*_m_;
(b) net reproductive value, *R*o;
(c) finite rate of increase, λ; and
(d) mean generation time, *T*.

The intrinsic growth rate (*r*_m_) was calculated using the formula of Wyatt and White [79]: *r*_m_ = 0,738 [(ln*M*_d_)/*d*], where *d* is the development period from birth to beginning of first reproduction and *M*_d_ is the number of nymphs born in the period from time d. Rearing was performed in a controlled environment of a growth chamber at 22–23 °C, 65% relative humidity, and light intensity of 130–150 μM photons m^−2^·s^−1^ with the 14/10 h (light/dark) photoperiod. For the calculation of demographic parameters used DEMOGRAF program [80].

### 4.4. The Effect of Nitric Oxide Donors on Behavioral Responses of *A. pisum* during Probing and Feeding

Experiments were conducted on 12-day-old pea seedlings pretreated with a specific NO donor, i.e., 200 μM GSNO/SNP, or not pretreated with GSNO/SNP (pretreated with H_2_O, control) in the light at 25 °C. Therefore, the whole pea seedlings growing on the hydroponic boxes containing Hoagland medium in glass boxes were thoroughly sprayed with a solution of GSNO/SNP or H_2_O (control) in the light. Next, for the influence of GSNO/SNP on the probing behavior of pea aphid *A. pisum*, apterous aphids were individually placed on pea seedling shoots, 24 or 48 h after pretreatment of seedlings with a specific NO donor or H_2_O. The effect of the application of GSNO/SNP to peas on the probing behavior of *A. pisum* was investigated by means of the Electrical Penetration Graph technique (EPG). This technique is commonly applied in Hemiptera-plant relationship studies [81,82]. The system is based on an electrical circuit where electrodes are attached to the aphid and the plant. When aphid stylets pierce into plant tissues, the circuit is completed. Aphid activities cause changes in electrical properties of the circuit and these changes are manifested as EPG waveforms. Presently, the meaning of most of the waveforms is known, so it is possible to relate them to specific aphid activities [83]. Based on the values derived from the analysis of various EPG parameters it is possible to assess the suitability of plants to aphids [53]. A 1 h starvation period after the attachment of the electrode is ensured before the start of the experiment. Each freshly prepared aphid/plant combination was used as an individual replicate. The experimental plants offered to the aphids were pretreated with GSNO or SNP for 24 and 48 h. A completely randomized design was used for these experiments. Giga-8 DC EPG system with a 1 GΩ of input resistance (EPG Systems, Wageningen, The Netherlands) was used to record EPGs. EPGs were recorded and analysed using Stylet+ release 2011_5 software (EPG Systems). The EPG recording started at 10–11 a.m. and was finished 24 h later. For analysis, the test plants pretreated for 24 and 48 h were labeled 24 h +SNP/24 h +GSNO and 48 h +SNP/48 h +GSNO, respectively. Various behavioral phases were labelled manually using the Stylet+ software. The analysis of EPG recordings included: np (non-probing, i.e., aphids with stylets outside plant tissues), C (pathway, i.e., probing in the epidermis and mesophyll and the so-called “derailed” stylet activities F and xylem sap consumption G), E1 (salivation into sieve elements), and E2 (phloem sap consumption). Several parameters related to the sequence and frequency of aphid activities during probing and stylet position in plant tissues were analysed [84]. Waveform patterns that were not terminated before the end of the experimental period (24 h) were included in the calculations. In sequential parameters, the duration of the period preceding the first phloem phase or first sustained ingestion phase equalled the time from the first probe until the end of the recording if either phase did not occur in a given aphid/VIP replicate. In sequential parameters, when time to waveforms related to phloem phase was calculated, if no phloem phase occurred, the time from the first probe until the end of the recording was used. In non-sequential parameters that described general aphid behavior, when a given waveform had not been recorded for an individual, the duration of that waveform was given the value “0” Phloem phase index was calculated as: phloem phase/phloem phase + non-phloem probing phase: E/E + C + F + G; was calculated as: phloem salivation/phloem phase: E1/E1 + E2. EPG parameters were calculated manually and individually for every aphid, and the mean and standard errors were subsequently calculated using the EPG analysis Excel worksheet created for this study. The Mann–Whitney U-test was applied to analyse differences in parameters derived from EPG recordings. The following comparisons were made: control: 24 h +SNP, control: 48 h +SNP, 24 h +SNP: 48 h +SNP and control: 24 h +GSNO, control: 48 h +GSNO, 24 h +GSNO: 48 h +GSNO.

### 4.5. Determination of Semiquinone Radicals and Manganese and Iron Ions

Radicals were detected directly in pea leaves using the electron paramagnetic resonance (EPR) technique [69,70]. Samples of 1 g fresh weight of pea leaves were frozen in liquid nitrogen and lyophilized in a Jouan LP3 freeze dryer. The lyophilized material was transferred to EPR-type quartz tubes (diameter 4 mm). Electron paramagnetic resonance was measured at room temperature with a Bruker ELEXSYS X-band spectrometer (Rheinstettenstate, Germany). The EPR spectra were recorded as first derivatives of microwave absorption. Microwave power of 2 mW and a 2 G magnetic field modulation were applied in all experiments to avoid signal saturation and deformation

EPR spectra were recorded for free radicals and Mn^2+^ and Fe^3+^ ions in the magnetic field range of 3300–3360 G and with 4096 data points. To determine the number of paramagnetic centres in the samples, the spectra were double-integrated and compared with the intensity of the standard Al_2_O_3_:Cr^3+^ single crystal with a known spin concentration [34,61,85,86,87,88,89,90]. Some background corrections of the spectra were introduced before and after the first integration to obtain a reliable absorption signal before the second integration. Concentrations of semiquinone radicals were calculated as the number of spins per 1 g of sample (dry weight/DW).

### 4.6. Determination of Superoxide Anion Content

The content of superoxide anion (O_2_^•–^) in biological samples was determined based on its ability to reduce nitro blue tetrazolium (NBT) according to the procedure described in [91] and modified by Mai et al. [61]. Leaves of pea (500 mg) were immersed in 10 mM potassium phosphate buffer (pH 7.8) containing 0.05% NBT and 10 mM NaN_3_, NADPH in a final volume of 3.5 mL and incubated for 1 h at room temperature. After incubation 2 mL of the reaction solution were heated at 85 °C for 15 min and rapidly cooled. The levels of O_2_^•−^ were expressed as absorbance at 580 nm per 1 g of fresh materials (A_580_·g^−1^ fr.wt.). A Perkin Elmer Lambda 15 UV-Vis spectrophotometer (Norwalk, CT, USA) was used in the analyses.

### 4.7. Analysis of Pisatin and Flavonols

#### 4.7.1. Isolation of Phenolic Compounds

Plant material, previouslyfrozen at −80 °C was homogenized in 80% methanol (20 mL·g^−**1**^·FW) and sonicated for 3 min in a VirTis VirSonic 60 sonicator [34]. The suspension was filtered through a Büchner funnel and concentrated under vacuum at 40 °C. Plant extract samples for LC analyses were prepared from 0.5 g FW pea tissue. The samples were purified and concentrated by solid-phase extraction on cartridges containing a cation exchanger and RP C-18 silica gel (Alltech, Carnforth, England, UK) used in tandem, according to the method proposed by Stobiecki et al. [92].

#### 4.7.2. Liquid Chromatography–Mass Spectrometry (LC/UV/ESI/MS/MS)

Plant extract samples were analysed using a Waters UPLC Acquity system coupled with a micrOToF-Q mass spectrometer (Bruker Daltonics, Bremen, Germany). An Agilent Poroshell RP-C18 column (100 × 2.1 mm; 2.7 µm) was used. During LC analyses elution was performed using with two solvent mixtures: A (95% H_2_O, 4.5% acetonitrile, 0.5% acetic acid; *v*/*v*/*v*) and B (95% acetonitrile, 4.5% H_2_O, 0.5% acetic acid; *v*/*v*/*v*). Elution steps were as follows: 0–5 min 10%–30% B, 5–12 min isocratic at 30% B, 12–13 min linear gradient up to 95% of B and 13–15 min isocratic at 95% of B. Pisatin and flavonols were identified by comparing their retention times and mass spectra with the data from respective standards. The micrOToF-Q mass spectrometer consisted of an ESI source operating at a voltage of ±4.5 kV, nebulization with nitrogen at 1.2 bar and dry gas flow of 8.0 L/min at 220 °C. The instrument was operated using the micrOTOF Control program version 2.3 and data were analyzed using the Bruker Data Analysis ver. 4 package. Targeted MS/MS experiments were performed using a collision energy ranging from 10 to 25 eV, depending on the molecular masses of compounds. The instrument operated at a resolution of minimum 15,000 full widths at half maximum.

For quantitative analysis, the extracted ion chromatogram traces from a MS detector were used, with peaks plotted for exact monoisotopic masses of compounds. *p*-Hydroxybenzoic acid was added to each analyzed sample as the internal standard at a final concentration of 125 µM (LC retention time and MS spectra did not interfere with those of the studied compounds).

### 4.8. Total RNA Extraction and Semiquantitative RT-PCR Analysis

Pea seedling leaves (0.50 g) were frozen in liquid nitrogen and ground with a mortar and pestle in the presence of liquid nitrogen. For RT-PCR analyses of the target gene, total RNA was isolated from 45 mg tissue using the SV Total RNA Isolation System (Promega, Manheim, Germany) according to the recommendations of the manufacturer [14,93]. The cDNA samples for RT-PCR experiments were synthesized from 1 μg of total RNA and oligo (dT)_18_ primers, using the High Capacity cDNA Reverse Transcription Kit (Applied Biosystems, Life Technologies Polska, Warszawa, Poland). One µL of reaction product (cDNA) served as a template for PCR reaction with specific PCR primers. Optimization of PCR reaction conditions (temperature and time of the individual steps, the number of cycles, the concentration of DNA polymerase, the concentration of Mg^2+^ and primers) was performed. Thermal cycling conditions in the PAL gene expression assay consisted of an initial denaturation at 95 °C for 5 min, followed by 25 cycles at 95 °C for 30 s, 58 °C for 30 s and 72 °C for 45 s. Standards, cDNA samples and the no-template control were analyzed in three replicates in each assay. The PCR products were analyzed by the agarose gel electrophoresis (1.5%) and the Gene Tools software, version 4.02 (Syngene). The specific primers for PAL were used for PCR reactions: the forward primer (F): 5′-CCAAGTCAATTGAGAGGGAG-3′ and the reverse primer (R): 5′-CATCTTGGTTGTGCTGCTC-3′. The fragment of *Pisum sativum* actin coding sequence was amplified as a reference gene using actin F (5′-GCATTGTAGGTCGTCCTCG-3′) and actin R (5′-TGTGCCTCATCACCAACATAT-3′) primers.

### 4.9. Extraction and Assay of β 1,3-Glucosidase Activity

The activity of β 1,3-glucosidase (EC 3.2.1.21) was determined spectrophotometrically (Perkin Elmer Lambda 15 UV-Vis spectrophotometer, Norwalk, CT, USA) applying the method proposed by Nichols et al. [94] and modified by Morkunas et al. [95]. Pea leaves (500 mg) were ground at 4 °C in 0.05 M phosphate buffer of pH 7.0 and 1% polyvinylpyrrolidone (PVP). The enzyme activity was determined in the supernatant obtained after centrifugation at 15,000× *g* for 20 min. The mixture containing 0.2 mL phosphate buffer (0.05 M, pH 7.0), 0.2 mL extract and 0.2 mL 4-nitrophenyl-b-d-glucopyranoside as substrate (2 mg·mL^−1^) was incubated for one hour at 35 °C. Afterwards, 0.6 mL 0.2 M Na_2_CO_3_ was added. The formation of *p*-nitrophenol (*p*-NP) was followed at 400 nm. The activity was measured in three replications and expressed as μM *p*-nitrophenol mg^−1^·protein·h^−1^.

### 4.10. Statistical Analysis

All determinations were conducted within three independent experiments. Analysis of variance (ANOVA) was used to verify significance of means from independent experiments within a given experimental variant. The ANOVA, StatSoft, Inc. (2009), STATISTICA, version 9.0, www.statsoft.com software was applied in statistical analysis of the data. The elementary comparisons between particular levels of the analyzed factor in different times (independently) were tested using the two-sample *t*-test for equal means for all observed traits. To account for multiple testing, we used the Bonferroni correction. Moreover, comparisons related to the following variants, i.e., variant +SNP/+GSNO vs. variant –SNP/−GSNO; variant −SNP/−GSNO vs. variant −SNP + aphid /−GSNO + aphid; variant +SNP/+GSNO vs. variant +SNP + aphid/+GSNO + aphid; variant −SNP + aphid/−GSNO + aphid vs. variant +SNP + aphid/+GSNO + aphid. The figures present data obtained as means of triplicates for each variant along with standard errors of mean (SE).

## 5. Conclusions

The data in the present work revealed the role of NO donors in the limiting of the *A. pisum* population growth rate. GSNO and SNP prolong the pre-reproductive period, limit the period of reproduction and have a significant impact on reducing fertility. Reduction of pea aphid performance was caused a consequence of reduced phloem sieve element contact. Results of these studies suggest sequence in the dynamic events that occur as a result of NO application and *A. pisum* infestation of *P. sativum*. In the presented study we revealed that NO induces a specified sequence of defense responses in *P. sativum*-*A. pisum* system. NO–induced defense strategy of *P. sativum* includes changes in free radicals generation and in the level of metal ions during *A. pisum* attack. Also, NO–induced changes in biosynthesis of flavonoids in this plant-aphid system. Results of research presented here will not only contribute novel information to our knowledge on plant biology but they may be of importance for the use of NO in pest control. However, more studies are required to elucidate the mechansism(s) by which NO donors enhance plant resistance to aphids.

## Figures and Tables

**Table 1:** Mean developmental time, longevity, total fecundity and rate of development of *A. pisum* on *P. sativum* seedlings exposed to pretreatment with an NO donor or with no pretreatment (control) (*n* = 20). Hypotheses on equality of means were verified by the two-sample *t*-test (Table S1).

Population and Life Parameters	Control	+GSNO	+SNP
Pre-reproductive period (days)	6.25 ± 0.44	7.25 ± 0.55	7.1 ± 0.55
Reproductive period (days)	23.25 ± 1.0	12.4 ± 2.76	11.0 ± 2.55
Post-reproductive period (days)	1.05 ± 1.14	0.90 ± 0.71	1.1 ± 0.71
Fecundity	172.7 ± 8.62	82.55 ± 6.50	91.6 ± 5.60
Longevity	30.55 ± 1.57	20.55 ± 2.89	19.2 ± 2.64

**Table 2:** Population and life table parameters of apterous *A. pisum* on *P. sativum* seedlings exposed to pretreatment with an NO donor or with no pretreatment (control). Ro, net reproductive rate; *r*_m_, intrinsic rate of increase; λ, finite rate of increase; *T*, generation time; DT, doubling time.

Variants	Parameter				
	*R*o	*r*_m_	λ	*T*	DT
Control	33.672	0.270	1.310	13.025	2.567
+GSNO	16.510	0.217	1.242	12.922	3.194
+SNP	18.322	0.229	1.257	12.699	3.027

**Table 3:** Mean (± SE) non-sequential and sequential electrical penetration graph (EPG) parameters describing the probing behavior of *A. pisum* on *P. sativum* L. treated with GSNO.

General Aspects of Aphid Probing Behavior	Control	24 h +GSNO	48 h +GSNO
	*n* = 19	*n* = 17	*n* = 17
Total duration (h) of non-probing phase np ^1^	2.3 ± 0.5	2.6 ± 0.6	5.5 ± 1.4
Total duration (h) of non-phloem probing phase C + G + F ^2^	8.4 ± 0.5	9.3 ± 0.8	10.2 ± 1.1
Total duration (h) of phloem phase E1 + E2 ^3^	13.2 ± 0.8	12.1 ± 1.1	8.3 ± 1.1b *^,†^
Phloem phase index ^4^	0.60± 0.03	0.55 ± 0.05	0.43 ± 0.05 *^,†^
Number of probes	53.8 ± 9.9	50.6 ± 9.6	61.2 ± 8.7
Number of probes with phloem phase	5.3 ± 0.4	5.4 ± 0.5	4.2 ± 0.7
Aphid probing behavior before 1st phloem phase	*n* = 19	*n* = 17	*n* = 15
Total duration (h) of non-probing before 1st phloem phase E1	0.7 ±0.03	1.1 ± 0.4	0.7 ± 0.2
Time to 1st phoem phase E1	2.1 ± 0.4	2.8 ± 0.8	6.1 ± 1.7 *^,†^
Time to 1st ingestion phase E2	2.1 ± 0.4	2.9 ± 0.8	6.1 ± 1.7 *^,†^
Time to 1st sustained (E2 > 10 min) ingestion phase	2.2 ± 0.4	2.9 ± 0.8	6.4 ± 1.7 *^,†^
Aphid probing behavior related to phloem phase	*n* = 19	*n* = 17	*n* = 15
Number of phloem phases E1 and E2	10.4 ± 1.3	11.4 ± 1.5	7.7 ± 1.4
Duration (h) of 1st phloem phase E1 + E2	1.2 ± 0.2	1.7 ± 0.5	1.7 ± 0.6
Phloem salivation index ^5^	0.02± 0.03	0.04 ± 0.08	0.05 ± 0.01

*n* = number of replications; in calculations referring to phloem phase only aphids that showed phloem phase were included; * difference in relation to control; ^†^ difference between 24GSNO and 48GSNO (Mann–Whitney-*U* test, *p* < 0.05); ^1^ Non-probing phase np: aphid stylets withdrawn from plant tissues; ^2^ Non-phloem probing phase includes pathway with cell punctures C, derailed stylet activities F, and xylem phase G; ^3^ Phloem phase includes salivation into sieve elements E1 and sap ingestion E2; ^4^ Index calculated as: duration of phloem phase E1 + E2/duration of phloem phase E1 + E2 + non-phloem probing phase C + F + G; ^5^ Index calculated as: duration of phloem salivation E1/duration of phloem phase E1 + E2.

**Table 4:** Mean (± SE) non-sequential and sequential electrical penetration graph (EPG) parameters describing the probing behavior of *Acyrthosiphon pisum* on *Pisum sativum* treated with SNP.

General Aspects of Aphid Probing Behavior	Control	24 h +SNP	48 h +SNP
	*n* = 10	*n* = 12	*n* = 12
Total duration (h) of non-probing phase np ^1^	9.4 ± 2.3	15.1 ± 2.1	13.6 ± 2.5
Total duration (h) of non-phloem probing phase C + G + F ^2^	7.2 ± 1.3	4.5 ± 0.8	5.2 ± 1.1
Total duration (h) of phloem phase E1 + E2 ^3^	7.4 ± 1.9	4.5 ± 2.0	5.2 ± 1.1
Phloem phase index ^4^	0.47 ± 0.07	0.32 ± 0.08	0.32 ± 0.09
Number of probes	62.6 ± 16.4	22.7 ± 4.0 *	31.8 ± 6.1
Number of probes with phloem phase	4.5 ± 0.8	2.3 ± 0.5 *	3.9 ± 1.0
Aphid probing behavior before 1st phloem phase	*n* = 10	*n* = 12	*n* = 12
Total duration (h) of non-probing before 1st phloem phase E 1	0.4 ± 0.2	1.1 ± 0.4	0.2 ± 0.1
Time to 1st phoem phase E1	1.3 ± 0.3	4.0 ± 1.1 *	4.2 ± 1.4
Time to 1st ingestion phase E2	1.3 ± 0.3	4.1 ± 1.1 *	4.2 ± 1.4
Time to 1st sustained (E2 > 10 min) ingestion phase	1.3 ± 0.3	4.7 ± 1.3 *	5.1 ± 1.5
Aphid probing behavior during phloem phase	*n* = 10	*n* = 12	*n* = 12
Number of phloem phases E1 and E2	8.0 ± 1.6	4.2 ± 1.1	5.8 ± 1.7
Duration (h) of 1st phloem phase E1 + E2	2.2 ± 0.7	1.2 ± 0.6 *	0.9 ± 0.5 *
Phloem salivation index ^5^	0.06 ± 0.01	0.09 ± 0.03	0.05 ± 0.02

*n* = number of replications; in calculations referring to phloem phase only aphids that showed phloem phase were included; * difference in relation to control; no difference between 24 h +SNP and 48 h +SNP was recorded (Mann–Whitney-U test, *p* < 0.05); ^1^ Non-probing phase np: aphid stylets withdrawn from plant tissues; ^2^ Non-phloem probing phase includes pathway with cell punctures C, derailed stylet activities F, and xylem phase G; ^3^ Phloem phase includes salivation into sieve elements E1 and sap ingestion E2; ^4^ Index calculated as: duration of phloem phase E1 + E2/duration of phloem phase E1 + E2 + non-phloem probing phase C + F + G; ^5^ Index calculated as: duration of phloem salivation E1/duration of phloem phase E1 + E2.

## Supplementary Materials

Supplementary materials can be found at www.mdpi.com/1422-0067/18/2/329/s1.

## Abbreviations

SNP	Sodium nitroprusside
GSNO	*S*-nitrosoglutathione
SNAP	*S*-nitroso-*N*-acetylpenicillamine
SIN-1	3-Morpholinosydnomine
+GSNO	Pretreated with GSNO and not infested by aphids
+SNP	Pretreated with SNP and not infested by aphids
−GSNO	Not pretreated with GSNO and not infested by aphids
−SNP	Not pretreated with SNP and not infested by aphids
+GSNO + aphid	Pretreated with GSNO and infested by aphids
+SNP + aphid	Pretreated with SNP and infested by aphids
−GSNO + aphid	Not pretreated with GSNO and infested by aphids
−SNP + aphid	Not pretreated with SNP and infested by aphids
NO	Nitric oxide
EPR	Electron paramagnetic resonance
fr. wt.	Fresh weight
dry wt.	Dry weight
g	Gram
g	The factor describing energy levels of the unpaired electron for the paramagnetic centre in an external magnetic field
h	Hour
hpi	Hours post infestation
ROS	Reactive oxygen species
O_2_^•−^	Superoxide anion
PAL	Phenylalanine ammonia-lyase
RNS	Reactive nitrogen species
ONOO^−^	Peroxynitrite
cADPR	Cyclic ADP ribose
Mn^2+^	Manganese ions
Fe^3+^	Iron ions
EPG	Electrical Penetration Graph
LC/UV/ESI/MS/MS	Liquid chromatography/ultraviolet detection/electrospray–mass spectrometry (tandem mass spectrometry)
24 h +GSNO	Seedlings offered to aphids 24 h after treatment with GSNO in EPG experiment
48 h +GSNO	Seedlings offered to aphids 48 h after treatment with GSNO in EPG experiment
24 h +SNP	Seedlings offered to aphids 24 h after treatment with SNP in EPG experiment
48 h +SNP	Seedlings offered to aphids 48 h after treatment with SNP in EPG experiment, PAMPs, Pathogen-associated molecular patterns, PTI, PAMP-triggered immunity; MAPK, Mitogen-activated protein kinase
