# Supplementary material for: The Dynamics of the Defense Strategy of Pea Induced by Exogenous Nitric Oxide in Response to Aphid Infestation
Source: Int J Mol Sci. 2017 Feb 5;18(2):329. doi: 10.3390/ijms18020329 (PMC5343865; doi:10.3390/ijms18020329)
Supplement: Supplementary file 1 [file ijms-18-00329-s001.pdf]

Supplementary Materials: The Dynamics of the Defense Strategy of Pea Induced by Exogenous Nitric Oxide in Response to Aphid Infestation

Agnieszka Woźniak, Magda Formela, Piotr Bilman, Katarzyna Grześkiewicz, Waldemar Bednarski, Łukasz Marczak, Dorota Narożna, Katarzyna Dancewicz, Van Chung Mai, Beata Borowiak-Sobkowiak, Jolanta Floryszak-Wieczorek, Beata Gabryś and Iwona Morkunas

Table S1. The elementary comparisons between particular levels of analyzed factor in different times (independently) using the two-sample *t*-test for equal means for demografic parameters.

Comparisons	Pre-Reproductive Period (Days)	Reproductive Period (Days)	Post-Reproductive Period (Days)	Fecundity	Longevity
Control v. GSNO	−7.43 **	12.55 ***	6.09 **	99.60 ***	22.39 ***
Control v. SNP	−8.03 **	14.80 ***	−2.81 *	88.67 ***	24.18 ***
GSNO v. SNP	1.31	1.55	−10.58 ***	−11.24 ***	1.57

* $p < 0.05$; ** $p < 0.01$; *** $p < 0.001$.

Table S2. The elementary comparisons between particular levels of analyzed factor in different times (independently) using the two-sample *t*-test for equal means for all observed traits.

Comparisons	Free Radical	Mn^{2+}	Fe^{3+}	Superoxide Anion	PAL Expression	Pisatin	Glc 2′OH Genistein	Glc-Glc-Glc-Rha Quercetin	Glc Isorhamnetin	β-glucosidase
−GSNO v. +GSNO (0 h)	0.72	−7.33 **	−11.72 ***	−5.77 **	−13.08 ***	−14.77 ***	15.67 ***	8.97 *	21.29 ***	−131.17 ***
−GSNO v. +GSNO (24 h)	−5.29 **	−7.65 **	−4.59 *	179.56 ***	14.43 ***	−32.51 ***	33.84 ***	47.89 ***	22.95 ***	−129.35 ***
−GSNO v. −GSNO + aphid (24 h)	−6.00 **	−18.92 ***	−6.05 **	31.91 ***	−58.34 ***	−3.90 *	1.10	−18.53 ***	−5.72 **	−73.80 ***
−GSNO + aphid v. +GSNO + aphid (24 h)	7.42 **	29.29 ***	8.06 **	−8.75 *	2.60	−0.6	1.56	40.69 ***	10.03 ***	89.03 ***
+GSNO v. +GSNO + aphid (24 h)	6.83 **	15.1 ***	6.61 **	−42.47 ***	−34.65 ***	31.76 ***	−8.44 **	−75.64 ***	−16.41 ***	131.86 ***
−GSNO v. +GSNO (48 h)	2.93 *	10.06 ***	6.38 **	−47.41 ***	−20.94 ***	0.43	2.87 *	7.16 **	−3.82 *	31.09 ***
−GSNO v. −GSNO + aphid (48 h)	10.63 ***	4.70 *	8.03 **	−2.93 *	−20.36 ***	3.55 *	0.89	2.98 *	−0.98	178.85 ***
−GSNO + aphid v. +GSNO + aphid (48 h)	−6.65 **	−1.15	−5.48 **	64.75 ***	20.24 ***	−40.10 ***	−0.73	−5.63 **	−2.00	−7.35 **
+GSNO v. +GSNO + aphid (48 h)	3.85 *	−2.38	−3.62 *	160.44 ***	21.00 ***	−68.99 ***	−3.11 *	−13.01 ***	1.20	29.46 ***
−GSNO v. +GSNO (72 h)	52.08 ***	12.00 ***	1.87	−15.00 **	6.67 **	56.21 ***	−4.96 **	−12.91 ***	4.18 *	18.91 ***
−GSNO v. −GSNO + aphid (72 h)	32.30 ***	−2.43	0.00	398.92 ***	9.81 ***	5.45 **	−2.41	−5.46 **	−20.59 ***	256.05 ***
−GSNO + aphid v. +GSNO + aphid (72 h)	−1.87	−0.16	−0.74	−109.22 ***	−3.50 *	19.37 **	−5.55 *	−7.53**	26.28 ***	−66.39 ***
+GSNO v. +GSNO + aphid (72 h)	−3.89 *	−17.45 ***	−1.96	54.56 ***	0.00	4.90 **	−1.14	0.53	−6.93 **	8.27 **
−SNP v. +SNP (0 h)	−8.45 **	0.69	3.99 *	75.01 ***	1.15	−28.84 ***	17.23 ***	10.28 **	6.96 **	−207.81 ***
−SNP v. +SNP (24 h)	−4.24 *	2.11	−0.36	163.22 ***	6.01 **	−31.30 ***	34.63 ***	46.27 ***	19.63 ***	−44.20 ***
−SNP v. −SNP + aphid (24 h)	−6.00 **	−18.92 ***	−6.05 **	31.91 ***	−27.56 ***	−3.90 *	1.10	−18.53 ***	−5.72 **	−73.80 ***
−SNP + aphid v. +SNP + aphid (24 h)	9.44 ***	14.88 ***	1.98	84.97 ***	−17.72 ***	1.67	4.34 *	6.76 **	27.61 ***	53.85 ***
+SNP v. +SNP + aphid (24 h)	6.40 **	−4.06 *	−7.00 **	−107.77 ***	−46.57 ***	35.49 ***	−15.26 ***	−28.01 **	0.14	33.63 ***
−SNP v. +SNP (48 h)	3.40 *	−1.68	2.33	−19.76 ***	−28.17 ***	41.27 ***	−2.85 *	−8.87 ***	6.68 **	38.34 ***
−SNP v. −SNP + aphid (48 h)	10.63 ***	4.70 *	8.03 **	−2.83 *	−9.64 ***	3.55 *	0.89	2.98 *	−0.98	178.85 ***
−SNP + aphid v. +SNP + aphid (48 h)	−0.87	−1.74	7.67 **	−22.16 ***	−8.7 ***	45.11 ***	−4.76 *	−4.76 **	−5.23 **	11.37 ***
+SNP v. +SNP + aphid (48 h)	8.37 **	13.72 ***	9.95 ***	−10.28 ***	3.29 *	−141.15 ***	2.62	4.71 **	−8.62 ***	14.83 ***
−SNP v. +SNP (72 h)	33.65 ***	−5.86 **	−0.39	20.47 ***	2.56	73.41 ***	−11.65 ***	−11.42 ***	6.62 **	−279.87 ***
−SNP v. −SNP + aphid (72 h)	32.30 ***	−2.43	0.00	398.92 ***	3.02 *	5.45 **	−2.41	−5.46 **	−20.59 ***	256.05 ***
−SNP + aphid v. +SNP + aphid (72 h)	−2.66	9.14 ***	1.46	−32.45 ***	14.72 ***	18.91 **	−2.05	−6.68 **	22.22 ***	−417.56 ***
+SNP v. +SNP + aphid (72 h)	−4.79 **	12.57 ***	1.95	−1.90	9.45 ***	−17.75 ***	4.7 **	−1.14	−21.19 ***	252.05 ***

* $p < 0.05$; ** $p < 0.01$; *** $p < 0.001$
